# Supplementary material for: Obesity and Diet Predict Attitudes towards Health Promotion in Pre-Registered Nurses and Midwives
Source: Int J Environ Res Public Health. 2021 Dec 20;18(24):13419. doi: 10.3390/ijerph182413419 (PMC8708316; doi:10.3390/ijerph182413419)
Supplement: Supplementary file 1 [file ijerph-18-13419-s001.zip › ijerph-1443264-supplementary.pdf]

International Journal of
Environmental Research and Public Health

MDPI

Supplementary File S1. Questionnaire items

Questionnaire measuring HPA

Please indicate your level of agreement/disagreement with the following statements by using the following options: strongly agree (4), agree (3), disagree (2), strongly disagree (1).

1. Nurses/midwives (as appropriate to your profession) should be role models for their patients by maintaining a healthy DIET.

2. Nurses/midwives (as appropriate to your profession) should be role models for their patients by maintaining a healthy WEIGHT.

3. I am confident that patients will value advice I give them regarding healthy eating.

4. I would feel embarrassed giving healthy eating advice to patients if I didn't follow it myself.

5. I would feel guilty giving healthy eating advice to patients if I didn't follow it myself.

6. I try not to advise people on a healthy diet

7. I use every opportunity to advise people on healthy diet

8. Patients would not respect healthy eating advice from a nurse/midwife (as appropriate to your profession) who is OVERWEIGHT/OBESE

9. It is hypocritical to promote healthy eating if you DO NOT EAT HEALTHILY

10. It is hypocritical to promote healthy eating if you are OVERWEIGHT/OBESE

11. My own DIET would not influence my discussions with patients about healthy eating

12. My own WEIGHT would not influence my discussions with patients about healthy eating

13. Patients are more likely to follow healthy eating advice from someone who looks like they eat healthily themselves

Health Promotion (HP) observation

1. I have witnessed nurses/midwives (as appropriate to your profession) promoting healthy eating on my placements so far.

Health Promotion (HP) engagement

1. I have personally engaged in promoting healthy eating on my placements

Role Attitude (RA) items

1. Promoting a healthy diet to patients is part of nurses'/midwives' (as appropriate to your profession) roles

2. Nurses/midwives (as appropriate to your profession) that give healthy eating advice should have a HEALTHY DIET themselves

3. Nurses/midwives (as appropriate to your profession) that give healthy eating advice should have a HEALTHY WEIGHT themselves
